# A1C Underperforms as a Diagnostic Test in Africans Even in the Absence of Nutritional Deficiencies, Anemia and Hemoglobinopathies: Insight From the Africans in America Study

**DOI:** 10.3389/fendo.2019.00533

**Published:** 2019-08-07

**Authors:** Sara M. Briker, Jessica Y. Aduwo, Regine Mugeni, Margrethe F. Horlyck-Romanovsky, Christopher W. DuBose, Lilian S. Mabundo, Thomas Hormenu, Stephanie T. Chung, Joon Ha, Arthur Sherman, Anne E. Sumner

**Affiliations:** ^1^Diabetes, Endocrinology, and Obesity Branch, National Institute of Diabetes, Digestive and Kidney Diseases, National Institutes of Health, Bethesda, MD, United States; ^2^National Institute of Minority Health and Health Disparities, National Institutes of Health, Bethesda, MD, United States; ^3^Laboratory of Biological Modeling Medicine, National Institute of Diabetes, Digestive and Kidney Diseases, National Institutes of Health, Bethesda, MD, United States

**Keywords:** diabetes, Africans, sickle cell trait, anemia, A1C

## Abstract

**Introduction:** To improve detection of undiagnosed diabetes in Africa, there is movement to replace the OGTT with A1C. The performance of A1C in the absence of hemoglobin-related micronutrient deficiencies, anemia and heterozygous hemoglobinopathies is unknown. Therefore, we determined in 441 African-born blacks living in America [male: 65% (281/441), age: 38 ± 10 y (mean ± SD), BMI: 27.5 ± 4.4 kg/m^2^] (1) nutritional and hematologic profiles and (2) glucose tolerance categorization by OGTT and A1C.

**Methods:** Hematologic and nutritional status were assessed. Hemoglobin <11 g/dL occurred in 3% (11/441) of patients and led to exclusion. A1C and OGTT were performed in the remaining 430 participants. ADA thresholds for A1C and OGTT were used. Diagnosis by A1C required meeting either A1C-alone or A1C&OGTT criteria. Diagnosis by OGTT-alone required detection by OGTT and not A1C.

**Results:** Hemoglobin, mean corpuscular volume and red blood cell distribution width were 14.0 ± 1.3 g/dL, 85.5 ± 5.3 fL, and 13.2 ± 1.2% respectively. B12, folate, and iron deficiency occurred in 1% (5/430), 0% (0/430), and 4% (12/310), respectively. Heterozygous hemoglobinopathy prevalence was 18% (78/430). Overall, diabetes prevalence was 7% (32/430). A1C detected diabetes in 32% (10/32) but OGTT-alone detected 68% (22/32). Overall prediabetes prevalence was 41% (178/430). A1C detected 57% (102/178) but OGTT-alone identified 43% (76/178). After excluding individuals with heterozygous hemoglobinopathies, the rate of missed diagnosis by A1C of abnormal glucose tolerance did not change (OR: 0.99, 95% CI: 0.61, 1.62).

**Conclusions:** In nutritionally replete Africans without anemia or heterozygous hemoglobinopathy, if only A1C is used, ~60% with diabetes and ~40% with prediabetes would be undiagnosed.

**Clinical Trial Registration:**: www.ClinicalTrials.gov, Identifier: NCT00001853

## Introduction

Undiagnosed diabetes in Africa has become an economic and public health challenge (1). In fact, 70% of Africans living with diabetes are undiagnosed (1). Historically, the OGTT has been the diagnostic standard for the detection of diabetes and its precursor state, prediabetes (2). However, due to cost and a shortage of health care professionals, conducting an OGTT is often not feasible (3). In 2010, the International Diabetes Federation (IDF) and the American Diabetes Association (ADA) approved hemoglobin A_1c_ (A1C) as a diagnostic alternative to the OGTT (4).

A1C is a measure of the degree to which hemoglobin A_1_ is glycated (3). Therefore, anemia and nutritional deficiencies which affect red blood cell physiology such as iron, B12, and folate compromise the performance of A1C (5). Furthermore, heterozygous hemoglobinopathies, specifically sickle cell trait (SCT) and hemoglobin C (HbC) trait, are common in Africa (6, 7). There is great uncertainty on whether the presence of a heterozygous hemoglobinopathy impacts the diagnostic efficacy of A1C (8–10).

Studies in mixed ancestry adults from South Africa have demonstrated that A1C detects abnormal glucose tolerance at a much lower rate than the 2-h glucose obtained from an OGTT (11). However, the degree to which iron, B12 or folate deficiency influenced the results of these studies is unknown (11).

By working with African-born blacks living in America, we address the ability of A1C to diagnose abnormal glucose tolerance in Africans who are neither anemic nor nutritionally compromised.

To use A1C or OGTT data to compile health statistics or establish healthcare plans in Africa, it is imperative to assess the reproducibility of diagnoses made by these tests. Therefore, we also address the reproducibility of diagnoses made by A1C and OGTT. In addition, as we are comparing the rate of detection of diabetes by A1C vs. OGTT, we also report on the severity of physiologic characteristics of diabetes when detection is made by A1C vs. by OGTT-alone.

This investigation had both primary and secondary goals. Our primary goals were to determine in African-born blacks living in America (1) nutritional and hematologic profiles and (2) glucose tolerance categorization by OGTT and A1C. Our secondary goals were (3) determine the diagnostic reproducibility of OGTT and A1C and (4) compare degree of glycemia, insulin resistance and beta-cell function in diabetes and prediabetes detected by A1C vs. that detected by OGTT and not A1C.

## Research Design and Methods Population

To assess diabetes and heart disease risk in African-born blacks living in the United States, the Africans in America cohort was established (12–15).

Recruitment was achieved by newspaper advertisements (45%), previous participant referrals (30%), NIH website and staff referrals (10%), community event outreach (10%), and flyers (5%). The study was approved by the National Institute of Diabetes Digestive and Kidney Diseases Institutional Review Board (Clinical Trials.gov Identifier: NCT00001853). Prior to enrollment, informed written consent was obtained.

To determine eligibility, telephone interviews were conducted. Prospective enrollees had to report that they were born in sub-Saharan Africa to two black parents who were also born in sub-Saharan Africa. In addition, they had to self-identify as healthy and state that to their knowledge they did not have diabetes.

After a telephone interview, 468 African-born blacks living in metropolitan Washington, DC came to the NIH Clinical Center for an outpatient screening visit. Thirty-nine individuals were excluded. Reasons for exclusion were anemia defined as hemoglobin <11 g/dL (*n* = 11), elevated liver transaminases (*n* = 1), declined blood draw (*n* = 1), pregnancy (*n* = 3), and scheduling conflicts (*n* = 20). In addition, two individuals were excluded because they had persistent fetal hemoglobin which interfered with A1c measurement. Hence, 430 individuals proceeded to Test Day 1.

The 430 participants were recruited in two consecutive waves: A and B (Supplement Figure 1).

Wave A consists of the first 332 enrolled individuals. At Test Day 1, A1C levels were measured and an OGTT (OGTT-1) performed. If the OGTT-1 met the ADA glucose criteria for diabetes, the person was invited to return to the Clinical Center for Test Day 2 for a repeat A1C and second OGTT (OGTT-2). Twenty-one individuals were diagnosed with diabetes and 18 agreed to return for a second A1C and OGTT-2.

Wave B consists of the 98 enrolled individuals who entered the study after Wave A. All Wave B participants were invited to the NIH Clinical Center for duplicate testing by A1C and OGTT. The two test days were 10 ± 7 days apart. Ninety-six of 98 enrollees agreed to be tested twice.

Therefore, OGTT-1 data are available for 430 participants (332 people from Wave A and 98 people from Wave B). OGTT-2 data were available for 114 (18 people from Wave A and 96 people from Wave B) (Supplement Figure 1).

## Outpatient Visits

### Screening Visit

A history, physical and electrocardiogram were performed. Routine blood tests were done to document the absence of anemia, kidney, liver, and thyroid disease.

### Test Day 1

Participants fasted for 12h and came to the Clinical Center at 7 a.m. Weight, height, waist circumference (WC), and blood pressure were obtained (12). A whole blood sample was obtained for A1C measurement and hemoglobin electrophoresis. An OGTT (Trutol 75; Custom Laboratories, Baltimore, MD) was performed with blood samples taken at baseline, 30, 60, and 120 min for determination of glucose and insulin concentrations.

After the OGTT was completed, a computerized tomographic (CT) scan (Siemens and Somatom Force Scanner, Munich, Germany) was performed at the level of the L2–3 vertebrae using automated software for the measurement of visceral adipose tissue (VAT) (12).

### Test Day 2

One hundred and fourteen participants returned to the NIH Clinical Center 10 ± 7 days after Test Day 1 for a repeat A1C and OGTT-2.

### Diagnosis of Glucose Tolerance Status

*A priori* it was decided that glucose tolerance category assignment was based on the results of Test Day 1.

   **Diabetes**: A1C ≥ 6.5%

                           or

                   FPG ≥ 126 mg/dL

                           or

                   2 h glucose ≥ 200 mg/dL.

   **Prediabetes:** 5.7% ≤ A1C ≤ 6.4%

                           or

                   100 mg/dL ≤ FPG < 126 mg/dL

                           or

                   140 mg/dL < 2 h glucose < 200 mg/dL

   **Normal glucose tolerance:**

   A1C<5.7% and FPG <100 mg/dL and 2 h glucose <200 mg/dL.

## Physiologic Measurements

### Degree of Glycemia

Area under the glucose curve using the trapezoidal rule (16):

$$\begin{array}{l} 0.5*fasting\;glucose+30\min glucose+1.5*60\min glucose\\ \;+120\min glucose \end{array}$$

### Insulin Resistance

Matsuda Index (17):

$$\begin{array}{l} \left(\frac{10,000}{\sqrt{fasting\;glucose\;\times fasting\;insulin\;\times mean\;glucose\;\times mean\;insulin}}\right). \end{array}$$

### Beta-Cell Function

The insulinogenic index (18):

$$\begin{array}{l} \left(\frac{30\min insulin-fasting\;insulin}{30\min glucose-fastingglucose}\right). \end{array}$$

## Assays

Hemoglobin, hematocrit, MCV, RDW, platelet count, and MPV were measured in EDTA-anticoagulated whole blood using a Sysmex XE-5000 analyzer (Chicago, IL). Glucose, insulin and liver enzymes were analyzed in serum with Roche Cobas 6000 analyzer (Roche Diagnostics, Indianapolis, IN). Vitamin B12 and folate in serum were analyzed with an Immulite 2000 XPi analyzer (Siemens Healthcare, Malvern, PA). Three hundred and seventy-nine consecutively enrolled individuals had albumin analyzed (Roche Cobas 6000 analyzer). Three hundred and ten consecutively enrolled individuals had reticulocyte count (Sysmex XE-5000 analyzer), and iron, transferrin, and ferritin were measured in serum (Roche Cobas 6000 analyzer).

### A1C by High Performance Liquid Chromatography

A1C values were measured by three different National Glycohemoglobin Standardization Program (NGSP)-certified instruments which were used sequentially by the NIH Clinical Center. All three instruments [BioRad Classic Variant (*n* = 32), Bio-Rad Variant II (*n* = 158) and BioRad D10 (*n* = 240)] were manufactured by BioRad Laboratories (Hercules, CA) and used similar HPLC technology. The correlation (R^2^) of the results between instruments was 99%.

### Hemoglobin Electrophoresis

Two hundred ninety-eight consecutively enrolled individuals had hemoglobin electrophoresis. However, 132 participants were enrolled before hemoglobin electrophoresis was included in the protocol. These 132 individuals had hemoglobin type determined by HPLC. Of these 132 participants, 7 had variant hemoglobin reported on their HPLC report but their HPLC tracing was unavailable. Therefore, the specific type of heterozygous hemoglobinopathy could not be determined (i.e., SCT, HbC trait, etc.). These individuals were classified as heterozygous hemoglobin-unknown type.

### Statistical Analyses

Unless stated otherwise, data are presented as mean ± SD. As appropriate, comparisons were made by unpaired t-tests, Mann-Whitney, one-way analyses of variance with Bonferroni corrections for multiple comparisons or chi-squared tests. Odds ratios were calculated with logistic regressions.

Reproducibility of diagnosis made by A1C and OGTT was determined with the κ-statistic and illustrated with Sankey plots [http://sankeymatic.com/build/ (19)]. The κ-statistic was interpreted according to standard criteria [slight (0–0.20), fair (0.21–0.40), moderate (0.41–0.60), substantial (0.61–0.80), excellent (0.81–1.0)] (20).

Study data were managed with Research Electronic Data Capture (REDCap) (21). *P-*values < 0.05 were considered significant. Analyses were performed with STATA (v 15, College Station, Texas).

## Results

Population characteristics are provided in Table 1. The African regions of origin of the participants were West (53%), Central (21%), and East (26%) (Table 1). The age of the participants was 38 ± 10 y. The age of entry into the United States was 26 ± 11 y. Years lived in the United States was 12 ± 9 y. West Africans lived in the United States the longest (*P* = 0.031).

**Table 1:** Metabolic and demographic characteristics.

Parameter^*^	**Total (*n* = 430)**	**West 53% (*n* = 229)**	**Central 21% (*n* = 89)**	**East 26% (*n* = 112)**	***P*-value^†^**
% Male	65%	64%	73%	62%	0.207
Age (y)	38 ± 10	39 ± 10	37 ± 11	38 ± 9	0.394
Age at immigration (y)	26 ± 11	26 ± 11	27 ± 9	26 ± 11	0.519
Years in the United States (y)	12 ± 9	13 ± 10	10 ± 8	11 ± 8	0.032, a^*^
BMI (kg/m^2^)	27.5 ± 4.4	27.7 ± 4.5	27.8 ± 5.0	26.7 ± 3.9	0.097
WC (cm)	90 ± 11	90 ± 12	89 ± 11	90 ± 11	0.782
VAT (cm^2^) (*n* = 420)	96 ± 68	92 ± 64	97 ± 73	102 ± 70	0.392
More than 1 drink/week (%)	42%	38%	54%	38%	0.030
Smoker (%)	4%	4%	3%	4%	0.910
Exercise IPAQ [(met min/wk)^*^10]	259 ± 277	249 ± 243	293 ± 306	260 ± 316	0.732
Married (%)	48%	46%	47%	54%	0.436
College graduate (%)	72%	75%	65%	75%	0.204
Health insurance (%)	68%	67%	66%	71%	0.676
Sickle cell trait (%)	13%	15%	18%	5%	0.013
Hemoglobin C trait (%)	3%	6%	0%	0%	0.002
Hemoglobin (g/dL)	14.0 ± 1.3	14.0 ± 1.4	14.0 ± 1.2	14.1 ± 1.4	0.537
Hematocrit (%)	41.8 ± 3.8	41.8 ± 4.0	41.6 ± 3.5	42.1 ± 3.5	0.593
MCV (fL)	85.5 ± 5.4	85.1 ± 5.8	84.8 ± 4.9	86.7 ± 4.4	0.013, b^*^, c^*^
RDW (%)	13.2 ± 1.2	13.3 ± 1.2	13.4 ± 1.1	13.0 ± 1.1	0.020, c^*^
Reticulocyte count (%) (*n* = 310)	1.47 ± 0.49	1.47 ± 0.52	1.50 ± 0.53	1.44 ± 0.43	0.711
B12 (pg/mL)	686 ± 410	759 ± 390	695 ± 348	531 ± 452	0.009, b^***^, c^*^
Folate (ng/mL)	13.1 ± 5.1	13.4 ± 5.0	13.4 ± 5.6	12.2 ± 4.6	0.092
Iron (mcg/dL) (*n* = 310)	88 ± 28	89 ± 28	92 ± 29	84 ± 27	0.233
Ferritin (mcg/L) (*n* = 310)	128 ± 106	135 ± 107	115 ± 87	125 ± 115	0.440
Albumin (*n* = 379)	4.0 ± 0.3	4.0 ± 0.3	4.0 ± 0.3	4.0 ± 0.2	0.014, b^*^
AST (U/L)	23 ± 13	24 ± 15	23 ± 9	21 ± 8	0.239
ALT (U/L)	27 ± 15	27 ± 16	30 ± 16	24 ± 13	0.006, c^**^
Creatinine (mg/dL)	0.91 ± 0.19	0.94 ± 0.20	0.92 ± 0.18	0.83 ± 0.17	0.009, b^***^, c^**^
A1C (%)	5.4 ± 0.7	5.4 ± 0.8	5.5 ± 0.5	5.3 ± 0.4	0.332
Fasting glucose (mg/dL)	92 ± 14	92 ± 16	91 ± 9	93 ± 9	0.675
2 h glucose (mg/dL)	132 ± 41	132 ± 44	132 ± 32	132 ± 43	0.999
AUC-glucose	541 ± 127	536 ± 137	547 ± 98	548 ± 125	0.615
Matsuda index (*n* = 423)	5.79 ± 3.88	5.74 ± 3.71	6.47 ± 5.03	5.36 ± 3.08	0.128
Insulinogenic index (*n* = 423)	1.74 ± 1.86	1.78 ± 1.73	1.62 ± 1.80	1.74 ± 2.14	0.791

* *Results presented as mean ± SD or percent*.

† *Comparison by One-Way ANOVA and chi-square for categorical variables*.

a *Comparison of West Africans to Central Africans*,

b *comparison of West Africans to East Africans*,

c *comparison of Central Africans to East Africans*.

* *P < 0.05*,

** *P < 0.01*,

*** *P < 0.001*.

### Body Size

BMI, WC and VAT did not vary by African region of origin (BMI: 27.5 ± 4.5 kg/m^2^, WC: 90 ± 11 cm and VAT: 95 ± 68 cm^2^).

### Social Factors

Central Africans reported a higher rate of alcohol consumption than West and East Africans (*P* = 0.030). However, smoking, exercise, marital status, education and health insurance did not differ by region.

### Hematologic and Nutritional Factors

SCT was more common in West and Central than East Africans (*P* = 0.013). HbC trait occurred only in West Africans.

Hemoglobin, hematocrit, iron concentrations, and related parameters (TIBC, % saturation and ferritin) did not vary by region. MCV was highest and RDW lowest in East Africans. B12, folate and iron deficiency occurred in 1% (5/430), 0% (0/430), and 4% (12/309) of participants, respectively. Albumin was <3.5 g/dL in 2% (9/379) of participants.

### Liver and Kidney Function

Liver function tests were either normal or less than 2SD above normal. Mean creatinine was 0.91 ± 0.19 mg/dL (range 0.52 to 1.95 mg/dL).

### Glycemia Measures

Neither glycemic parameters (A1C, fasting, and 2 h glucose) nor measurements of glucose physiology (AUC-glucose, insulin resistance, and beta-cell function) varied by African region. Therefore, the Africans from the three regions—West, Central, and East—were evaluated together.

### Diabetes Prevalence

The prevalence of previously undiagnosed diabetes was 7% (32/430).

**Figure 1:**
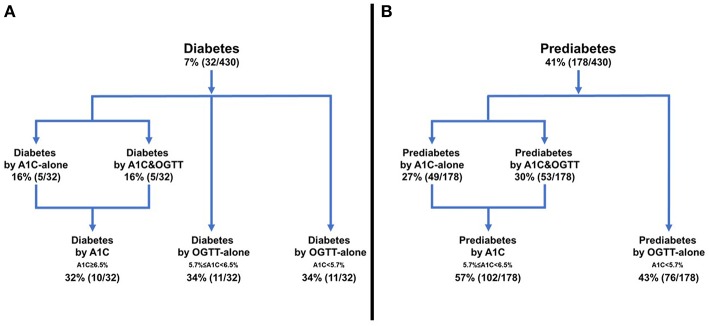
Frequency of diabetes or prediabetes according to diagnostic test. **(A)** Diabetes, **(B)** Prediabetes.

People with diabetes were divided into 3 groups (Figure 1A):

1. Diabetes by A1C (A1C-alone or A1C&OGTT), 32% (10/32);
2. Diabetes by OGTT-alone but prediabetes by A1C, 34% (11/32);
3. Diabetes by OGTT-alone but NGT by A1C, 34% (11/32).

**Figure 2:**
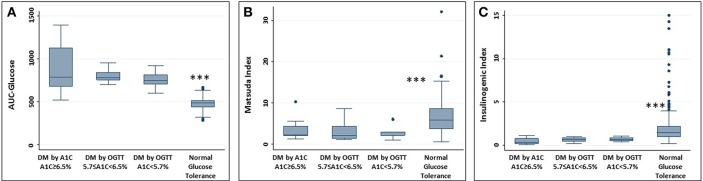
Measures of glucose physiology in individuals with diabetes. Normal glucose tolerance group provided for reference. **(A)** AUC-glucose; **(B)** Matsuda index; **(C)** Insulinogenic index. No difference between the 3 groups with diabetes. Difference between each group with diabetes and NGT, ^***^P < 0.001.

Degree of glycemia (AUC-glucose), insulin resistance (Matsuda index), and beta-cell function (insulinogenic index) did not differ by diabetic group (all *P* > 0.3) but were significantly different from the NGT group (*P* < 0.001) (Figure 2).

Age, BMI, WC, and VAT did not differ by diagnostic modality. In addition, liver and kidney function, prevalence of SCT and hemoglobin did not vary. Therefore, these potential confounders do not explain the differences in detection of diabetes by A1C vs. OGTT-alone (Supplement Table 1).

### Prediabetes Prevalence

The prevalence of prediabetes in the cohort was 41% (178/430). The 178 people with prediabetes were divided into 2 groups (Figure 1B):

1. Prediabetes detected by A1C (A1C-alone or A1C&OGTT), 57% (102/178);
2. Prediabetes detected by OGTT-alone but NGT by A1C, 43% (76/178).

### Relationship of Heterozygous Hemoglobinopathy to A1C Levels

For people with both diabetes and prediabetes, A1C levels did not differ by hemoglobin type (Supplement Figure 2). For example, A1C levels in diabetes and hemoglobin type AA vs. heterozygous hemoglobinopathy were 6.7 ± 1.7 vs. 5.7 ± 0.9, *P* = 0.120. For prediabetes, A1C levels in hemoglobin type AA vs. heterozygous hemoglobinopathy were 5.6 ± 0.4 vs. 5.6 ± 0.5, *P* = 0.581.

### Diabetes

Of the 19 participants with diabetes by OGTT and hemoglobin type AA, 63% (12/19) had A1C < 6.5% (Supplement Table 2A). Therefore, 63% of people with diabetes and hemoglobin type AA would not be detected if only A1C was obtained.

Of the 13 individuals with both diabetes and heterozygous hemoglobinopathy, 77% (10/13) had A1C < 6.5% (Supplement Table 2B). Therefore, 77% of people with diabetes and heterozygous hemoglobinopathy would not be detected if only A1C was obtained.

### Prediabetes

For the participants with prediabetes and hemoglobin type AA, 43% (65/152) had A1C < 5.7%. Therefore, 43% of people with prediabetes and hemoglobin type AA would not be detected if only A1C was obtained.

Of the 25 people with both prediabetes and heterozygous hemoglobinopathy, 40% (10/25) had A1C <5.7%. Therefore, 40% would not be detected if only A1C was obtained.

Overall, heterozygous hemoglobinopathies did not influence the odds of underdiagnosis of abnormal glucose tolerance by A1C (OR: 0.99, 95% CI: 0.61, 1.62).

### Reproducibility

One hundred and fourteen individuals had duplicate OGTT 10 ± 7 days apart. One participant did not have a repeat A1C on the day of their second OGTT.

### Reproducibility of Diagnoses Made by OGTT

**Figure 3:**
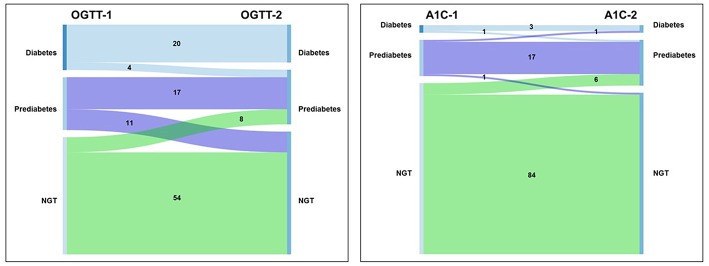
Sankey diagram of transitions between glucose tolerance categories. **(A)** OGTT; **(B)** A1C. Left side of each panel is the proportion of individuals with diagnosis at the first test. Right side of each panel is the proportion of individuals with diagnosis at the second test.

For the binary outcome of diabetes or no diabetes, reproducibility was excellent (κ = 0.89). Of note, no one without diabetes on Test Day 1 was diagnosed with diabetes on Test Day 2 (Figure 3A).

Excluding people with diabetes and considering the binary outcome of prediabetes or NGT, reproducibility of the diagnosis of prediabetes declined to moderate (κ = 0.49). This relatively low degree in reproducibility is largely accounted for by movement between NGT and prediabetes for people with fasting glucose ≥90 mg/dL. Individuals with fasting plasma glucose (FPG) ≥90 mg/dL were 2.5 times more likely to transition between prediabetes and NGT than people with FPG <90 mg/dL [OR 2.58 (95% CI: 1.31, 5.06), *P* = 0.006].

### Reproducibility of Diagnoses Made by A1C

For the diagnosis of the presence or absence of diabetes, reproducibility was substantial (κ = 0.65). One person without diabetes on Test Day 1 was diagnosed with diabetes on Test Day 2 (Figure 3B).

Similarly, for the diagnosis of prediabetes, reproducibility was substantial (κ = 0.65).

## Discussion

Working with African-born blacks living in America, we are the first to report that even in the absence of nutritional and hematological confounders, ~60% of Africans with diabetes and ~40% with prediabetes would be undiagnosed if A1C replaced the OGTT. This level of underperformance of A1C is within the 20 to 80% lower range of detection of diabetes observed in whites, mixed ancestry populations, indigenous peoples, Asians, and Hispanics (11, 22, 23).

It is speculated that the poor performance of A1C in Africans can be accounted for by undiagnosed anemia, hemoglobin-related micronutrient deficiencies, liver or kidney disease or heterozygous hemoglobinopathies (24, 25). However, we were able to screen for each of these conditions and document that in the Africans in America cohort, these factors did not account for underperformance of A1C as a diagnostic test.

### Detection of Diabetes by OGTT but Not A1C

Of the people diagnosed with diabetes by OGTT-alone, A1C classified one half with prediabetes and one half with NGT. Concern needs to be focused on individuals diagnosed with diabetes but identified as NGT by A1C. Due to the A1C diagnosis of NGT, two adverse events occur. First, the prevalence of diabetes is underestimated. Second, healthcare providers do not provide to these patients' critical information on diet, medication, lifestyle or eye or foot care. In short, lack of the correct diagnosis increases the risk for progressive, preventable disease (26).

### Detection of Prediabetes by OGTT but Not A1C

We found that if only A1C was performed, 43% of individuals with prediabetes would be categorized as NGT by A1C. This is problematic because even a single abnormal OGTT is an important risk factor for diabetes and cardiovascular disease (27). Furthermore, the evidence that conversion from prediabetes to diabetes can be prevented or delayed by lifestyle interventions is based on OGTT diagnosed disease (28).

### Physiologic Severity

We performed multi-sampled OGTT and calculated the degree of glycemia (AUC-glucose), insulin resistance (Matsuda Index), and β-cell function (insulinogenic index) in diabetes detected by A1C vs. OGTT-alone. Hence, we are able to report that the physiologic severity of diabetes and prediabetes detected by A1C vs. OGTT-alone is equivalent. Therefore, abnormal glucose tolerance diagnosed by either test should be treated.

### Heterozygous Hemoglobinopathy

There is concern that heterozygous hemoglobinopathies might contribute to the underperformance of A1C (8, 9). However, in our study, the presence of heterozygous hemoglobinopathies did not increase the odds of under detection of abnormal glucose tolerance by A1C. Therefore, we do not attribute the poor performance of A1C as a diagnostic test to the presence of heterozygous hemoglobinopathies.

However, there may be two reasons for the difference in our assessment of the effect of heterozygous hemoglobinopathy. First, we were able to operate under more optimal conditions. First, people with anemia were excluded. Second, we were able to document that <5% of participants had low B12, folate or iron concentrations. Furthermore, in an earlier study of 90 participants enrolled in this cohort, we analyzed A1C by both HPLC (BioRad) and boronate affinity chromatography (10). Twenty-three percent (22/90) of that sample had heterozygous hemoglobinopathy. Agreement between A1C levels determined by the two methods, boronate affinity and HPLC, in both groups was excellent. In short, under ideal conditions heterozygous hemoglobinopathies do not appear to interfere with determination of A1C levels. But ideal conditions are not the norm in clinical practice.

### Reproducibility of Diagnosis

We evaluated OGTT reproducibility by analyzing diabetes and prediabetes separately. When the analysis was designed to determine the presence or absence of diabetes, reproducibility was excellent (κ = 0.89). This high degree of reproducibility is consistent with the observation that no one transitioned from diabetes to NGT and no new cases of diabetes were detected at the second OGTT. In short, the high degree of reproducibility for the diagnosis of diabetes in Africans should engender confidence in a diagnosis made by a single OGTT.

However, when the diagnosis was confined to the presence or absence of prediabetes, the OGTT exhibited only moderate reproducibility (κ = 0.49). This degree of reproducibility of the diagnosis by prediabetes by OGTT is consistent with studies in other populations (27, 29). Overall, low reproducibility for the diagnosis of prediabetes by the OGTT is to be anticipated because prediabetes is a transitory state. We found that those in the subgroup with fasting glucose between 90 and 100 mg/dL were most likely to transition between NGT and prediabetes.

Reproducibility of diagnosis by A1C was substantial for both diabetes and prediabetes (κ = 0.65). However, as the overall rate of detection of diabetes and prediabetes was so low at the first study, serial testing with A1C is not likely to resolve the problem of underdiagnosis.

### Strengths and Limitations

A major strength of this study was our access to a wide range of data, including (a) multi-sampled OGTT (b) duplicate studies in 114 participants, (c) hemoglobin electrophoresis and (d) blood work, which made it possible to rule out A1C confounders such as anemia, iron, folate and B12 deficiencies as well as liver and kidney dysfunction.

There are also weaknesses. We recognize that our sample size is relatively small. However, the prevalence of diabetes in our cohort was 7%, which is similar to the prevalence of diabetes in African-born blacks living in Canada (30). Equivalent epidemiologic data is not available in the United States. In addition, our sample size was large enough that the prevalence of sickle cell trait was significantly higher in West and Central Africa than East Africa. Correspondingly, the mean corpuscular volume (MCV) was lower in West and Central than East Africa.

Although this was a cross-sectional study, our goal was to determine the prevalence of diabetes (and prediabetes) if A1C replaced the OGTT. Nonetheless, it would be ideal to have a prospective study to compare disease progression in A1C diagnosed disease vs. OGTT-alone diagnosed disease.

## Conclusions

Our investigation confirms that A1C underperforms as a diagnostic test for hyperglycemic states even in the absence of nutritional deficiencies or hemoglobinopathies. Therefore, we agree that while controversy exists in the diagnostic efficacy of A1C overall, as a single test in Africans, A1C is not an optimal substitute for the OGTT (11, 24, 31, 32). In short, the poor diagnostic performance of A1C as a single test in African-born blacks has the potential to contribute to an underestimation of diabetes prevalence and compromise healthcare resource allocation and care. However, emerging data suggests there may be value in combining A1C with other tests such as FPG or glycated albumin (10, 33, 34). This is important because the OGTT cannot be recommended because it is neither cost-effective nor feasible. Emerging alternatives to the OGTT include fasting glucose, glycated albumin, fructosamine, and 1,5-anhydroglucitol (10, 33, 35). Optimizing the diagnosis of glucose tolerance status in Africans requires investment in research to assess if these alternatives alone or in combination with A1C could be the way forward.

## Data Availability

The datasets generated for this study are available on request to the corresponding author.

## Ethics Statement

This study was approved by National Institute of Diabetes Digestive and Kidney Diseases Institutional Review Board (www.ClinicalTrials.gov Identifier: NCT00001853). Prior to enrollment, informed written consent was obtained.

## Author Contributions

SB, JA, CD, LM, SC, and AES collected the data. SB, JA, RM, MH-R, TH, SC, JH, AS, and AES analyzed the data. SB and AES wrote the manuscript. SB, JA, RM, MH-R, CD, LM, TH, SC, JH, AS, and AES provided critical rewrites.

### Conflict of Interest Statement

The authors declare that the research was conducted in the absence of any commercial or financial relationships that could be construed as a potential conflict of interest.
