# Supplementary material for: A1C Underperforms as a Diagnostic Test in Africans Even in the Absence of Nutritional Deficiencies, Anemia and Hemoglobinopathies: Insight From the Africans in America Study
Source: Front Endocrinol (Lausanne). 2019 Aug 7;10:533. doi: 10.3389/fendo.2019.00533 (PMC6692432; doi:10.3389/fendo.2019.00533)
Supplement: Supplementary file 1 [file Table_1.DOCX]

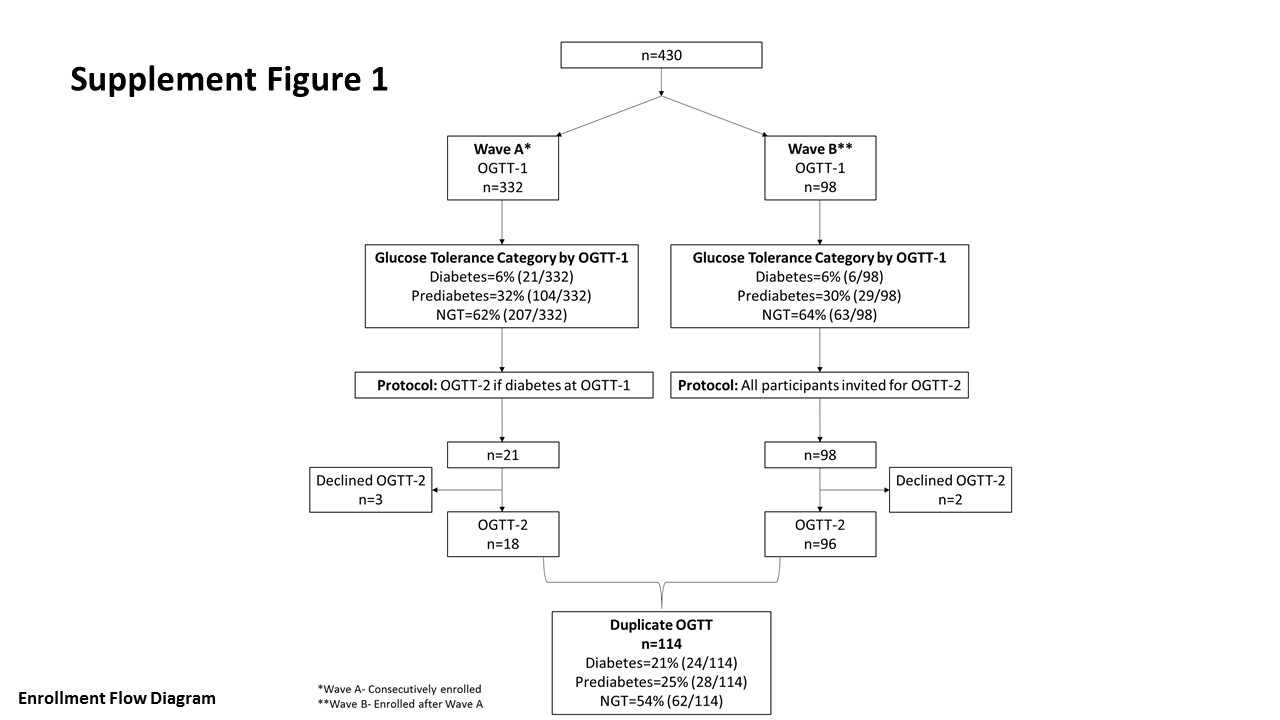


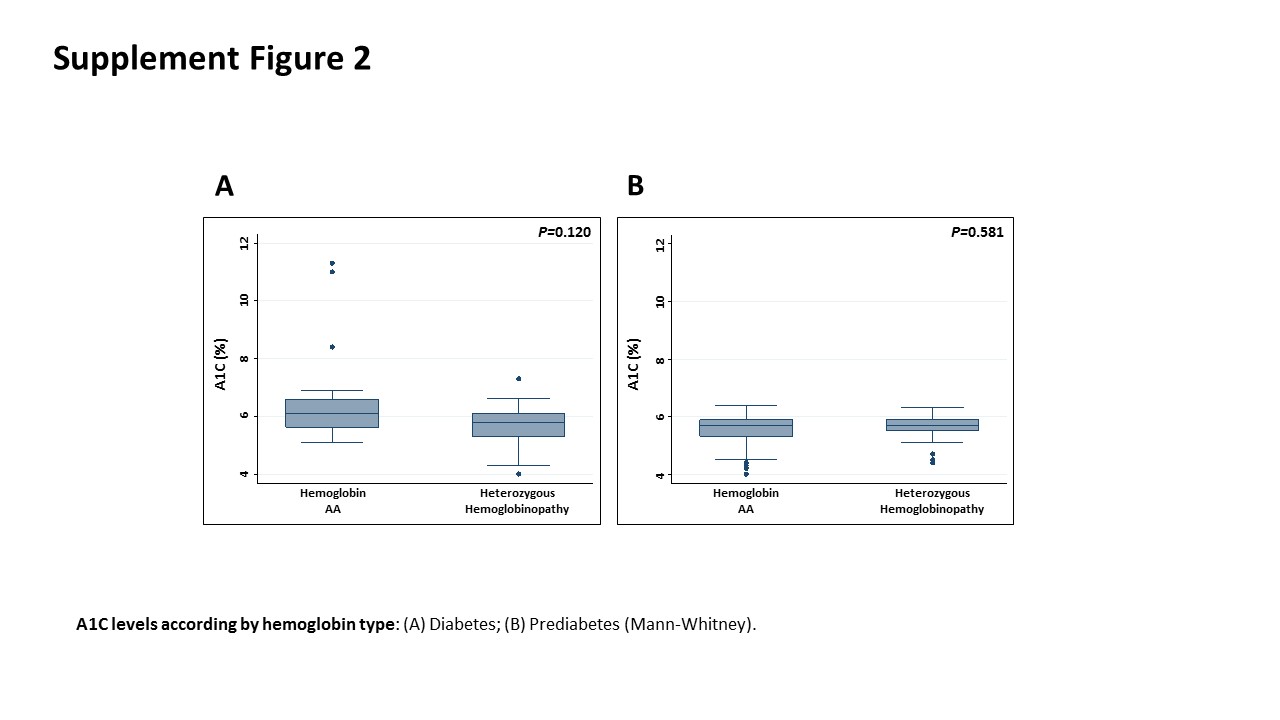


**Supplement Table 1: Characteristics of Participants with Diabetes**

| **Parameter^1^** | **Diabetes**  **Dx byA1C**  **A1C (≥6.5%)**  **n=10** | **Diabetes**  **Dx by OGTT**  **A1C (5.7 to 6.4%)**  **n=11** | **Diabetes**  **Dx by OGTT**  **A1C (<5.7%)**  **n=11** | ***P*-value^2^** |
| --- | --- | --- | --- | --- |
| **AUC-Glucose** | 890±301 | 807±83 | 762±99 | 0.291 |
| **Matsuda Index** | 3.61±2.73 | 3.05±2.01 | 3.18±1.64 | 0.847 |
| **Insulinogenic Index** | 0.49±0.42 | 0.63±0.25 | 0.69±0.22 | 0.315 |
| **A1C (%)** | 7.8±1.9 | 6.1±0.2 | 5.1±0.5 | <0.001, a**; b*** |
| **Fasting glucose (mg/dL)** | 139±52 | 109±18 | 105±11 | 0.066 |
| **2h glucose (mg/dL)** | 238±92 | 241±28 | 225±24 | 0.784 |
| **Age (y)** | 44±10 | 48±10 | 40±8 | 0.120 |
| **BMI (kg/m^2^)** | 30.5±6.3 | 29.2±4.7 | 28.9±3.9 | 0.749 |
| **WC (cm)** | 98±16 | 102±13 | 98±9 | 0.785 |
| **VAT (cm^2^)** | 167±107 | 151±42 | 150±63 | 0.862 |
| **AST (U/L)** | 30±20 | 23±10 | 32±28 | 0.589 |
| **ALT (U/L)** | 37±24 | 34±24 | 37±16 | 0.917 |
| **eGFR (mL/min/1.73m^2^)^3^** | 97±24 | 111±24 | 107±24 | 0.413 |
| **SCT (%)** | 30% | 36% | 27% | 0.895 |
| **Hgb (g/dL)** | 13.8±1.3 | 14.3±1.3 | 14.5±1.6 | 0.476 |

^1^Results presented as mean±SD

^2^ Comparison by one-way ANOVA: a: Column 1 vs. Column 2; b: Column 1 vs. Column 3; c: Column 2 vs. Column 3 * *P*<0.05; ***P*<0.01; ****P*<0.001

^3^MDRD Equation

**Supplement Table 2: Participants with Diabetes rank-ordered by A1C: (A) Hemoglobin type AA; (B) Heterozygous Hemoglobin**

**A B**

|  | **Birth Country** | **A1C (%)** | **Hb Type** | **Diabetes Diagnosed**  **by A1C** |  |  | **Birth Country** | **A1C (%)** | **Hb**  **Type** | **Diabetes Diagnosed**  **by A1C** |
| --- | --- | --- | --- | --- | --- | --- | --- | --- | --- | --- |
| 1 | Ghana | 11.3 | AA | Yes |  | 1 | Ghana | 7.3 | AS | Yes |
| 2 | Nigeria | 11.0 | AA | Yes |  | 2 | Liberia | 6.6 | AS | Yes |
| 3 | Ghana | 8.4 | AA | Yes |  | 3 | Nigeria | 6.5 | AS | Yes |
| 4 | Ghana | 6.9 | AA | Yes |  | 4 | Ghana | 6.1 | AS | No |
| 5 | DRC* | 6.6 | AA | Yes |  | 5 | Cameroon | 6.2 | AS | No |
| 6 | DRC* | 6.6 | AA | Yes |  | 6 | Nigeria | 6.0 | AS | No |
| 7 | Cameroon | 6.5 | AA | Yes |  | 7 | Ghana | 5.8 | AS | No |
| 8 | Rwanda | 6.4 | AA | No |  | 8 | Nigeria | 5.5 | AS | No |
| 9 | Rwanda | 6.3 | AA | No |  | 9 | Uganda | 5.4 | AS | No |
| 10 | Cameroon | 6.1 | AA | No |  | 10 | Uganda | 5.3 | AS | No |
| 11 | Ethiopia | 6.0 | AA | No |  | 11 | Ghana | 5.2 | AC | No |
| 12 | Ethiopia | 6.0 | AA | No |  | 12 | Ghana | 4.3 | AC | No |
| 13 | Cameroon | 5.9 | AA | No |  | 13 | Uganda | 4.0 | HH^2^ | No |
| 14 | Gambia | 5.9 | AA | No |  | ^1^Heterozygous hemoglobinopathy type: Unknown | | | | |
| 15 | Uganda | 5.6 | AA | No |  |  |  |  |  |  |
| 16 | Somalia | 5.5 | AA | No |  |  |  |  |  |  |
| 17 | Nigeria | 5.4 | AA | No |  |  |  |  |  |  |
| 18 | Cameroon | 5.3 | AA | No |  |  |  |  |  |  |
| 19 | DRC^1^ | 5.1 | AA | No |  |  |  |  |  |  |
| *Democratic Republic of the Congo | | | | |  |  |  |  |  |  |
|  |  |  |  |  |  |  |  |  |  |  |
